# Supplementary material for: Causal Relationship between Aging and Anorexia Nervosa: A White-Matter-Microstructure-Mediated Mendelian Randomization Analysis
Source: Biomedicines. 2024 Aug 16;12(8):1874. doi: 10.3390/biomedicines12081874 (PMC11351342; doi:10.3390/biomedicines12081874)
Supplement: Supplementary file 1 [file biomedicines-12-01874-s001.zip › Supplementary Figure/Figure S8/Leaveoneout plot of MR analysis for TL to ubm-b-2097.pdf]

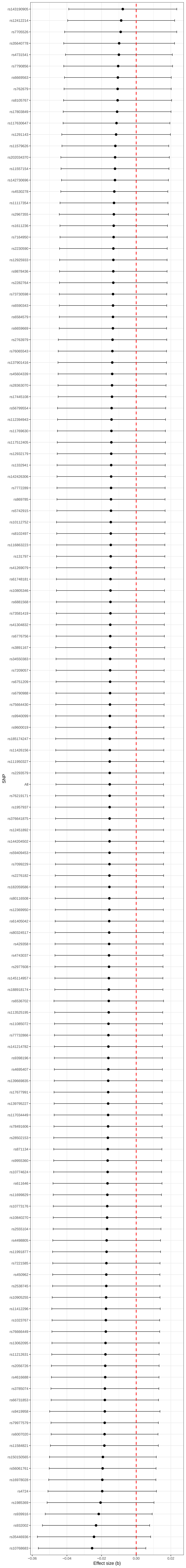
rs143190905
rs12412214
rs7705526
rs35640778
rs4731541
rs7790856
rs6669563
rs762679
rs8105767
rs17803849
rs117630647
rs1291143
rs11579626
rs202034370
rs11557154
rs142730696
rs4530278
rs11117354
rs2967355
rs1611236
rs7164950
rs2230590
rs12925933
rs9878436
rs2282764
rs73730598
rs6590343
rs6584579
rs6659669
rs2763979
rs76065543
rs137901416
rs45604339
rs28363070
rs17445108
rs56799554
rs112394943
rs11769630
rs117512405
rs12932179
rs1332941
rs142426306
rs7772289
rs869785
rs5742915
rs10112752
rs8102497
rs116863223
rs131797
rs41269079
rs61748181
rs10805346
rs6881568
rs73581419
rs41304832
rs6776756
rs3891167
rs34550383
rs7209057
rs6751209
rs6790988
rs75664430
rs9940099
rs9600019
rs185174247
rs11426156
rs111950327
rs2293579
All
rs76219171
rs1957937
rs376641875
rs12451892
rs144204502
rs59409453
rs7099229
rs2276182
rs182059586
rs80116508
rs12369950
rs61405042
rs80324517
rs429358
rs4743037
rs2977608
rs145114957
rs188918174
rs6536702
rs113525195
rs11085072
rs77732866
rs141214782
rs9398196
rs4695407
rs139669835
rs17677991
rs139795227
rs117034449
rs78491606
rs28502153
rs871134
rs9955360
rs10774624
rs611646
rs11699829
rs10773176
rs10840270
rs2555104
rs4498805
rs11991877
rs7221585
rs450962
rs2538745
rs10905255
rs11412296
rs1023767
rs76666449
rs13062095
rs11212631
rs2056726
rs4616688
rs3785074
rs66731853
rs9419958
rs79977579
rs6007020
rs11584821
rs150150565
rs56061761
rs16978028
rs4724
rs1985369
rs939916
rs932002
rs35446936
rs10768683
SNP
−0.06
−0.04
−0.02
0.00
0.02
Effect size (b)
